# A Dendrimer-Based Multiple Antigenic Peptide (MAP) Approach for Dengue Vaccine Development: In Silico and In Vivo Insights on Safety and Effectiveness

**DOI:** 10.3390/biology15141201

**Published:** 2026-07-20

**Authors:** Amtul Wadood Wajeeha, Najam us Sahar Sadaf Zaidi, Deeba Amraiz, Naseeha Bibi, Mamuna Mukhtar, Sobia Asghar, Muhammad Tahir

**Affiliations:** 1 Atta ur Rahman School of Applied Biosciences (ASAB), National University of Sciences and Technology (NUST), Islamabad 44000, Pakistan; awwajiha81@gmail.com (A.W.W.); naseehaqureshi@gmail.com (N.B.); mamunakhan26@gmail.com (M.M.); sobia.asghar64@gmail.com (S.A.); 2 Department of Biological and Health Sciences, Pak-Austria Fachhochschule Institute of Applied Science and Technology, Haripur 22620, Pakistan; sadaf.zaidi@paf-iast.edu.pk; 3 Department of Biological Sciences, National University of Medical Sciences, Rawalpindi 46000, Pakistan; deeba.amraiz@numspak.edu.pk

**Keywords:** dengue virus, multiple antigenic peptide (MAP), vaccine development, ELISA, MTT assay, histopathology

## Abstract

Dengue fever, a rapidly spreading mosquito-borne viral disease, poses a major global health issue, particularly in tropical and subtropical regions. The absence of a universally effective vaccine against all four dengue virus serotypes necessitates continued exploration of novel vaccine strategies. This study evaluated a PrM-derived multiple antigenic peptide (MAP) vaccine candidate in BALB/c mice. Molecular docking analyses predicted favourable interactions of the selected PrM-derived B-cell epitope with both human (human BCR Fab region PDB ID: 5IFH) and mouse (mouse BCR PDB ID: 8EMA) B-cell receptors, supporting its antigenic potential. ChemSketch was used to develop the PrM–MAP vaccine construct, which was then commercially synthesized. Mice received single, double, and triple booster doses of the vaccine, while control groups received either PBS or adjuvant alone. Humoral immune responses were measured by ELISA, and safety was evaluated through in vitro cytotoxicity assays on HEK293 cells and the in vivo histopathological examination of major organs. Mice immunized with a single booster dose produced significant levels of PrM-specific antibodies compared to subsequent booster doses, suggesting variability in the humoral response elicited. Optimal coating concentrations in ELISA demonstrated the importance of antigen concentrations in the sensitivity of the assay. The safety evaluation indicated high cell viability, up to 150 µg/mL of PrM–MAP, in HEK293 cells, and an absence of significant histopathological damage in treated animals. The PrM–MAP vaccine construct elicited a detectable humoral immune response while demonstrating a high safety profile in vitro and in vivo. These findings indicate the potential of MAP-based vaccine platforms as promising candidates for further development against the dengue virus.

## Simple Summary

Dengue fever remains one of the biggest health challenges for tropical and subtropical countries. The WHO has licensed two vaccines, Dengvaxia^®^ and TV003/TV005, to prevent the infection. Still, there are concerns about the efficacy of those vaccines and the emergence of antibody-dependent enhancement. This research aimed to develop a multiple antigenic peptide (MAP) vaccine that uses a dendrimer structure, containing four copies of a PrM-derived B-cell epitope previously selected via an in silico approach. It eliminates the risk of developing allergies, toxicity, and ADE while maintaining its antigenic and immunogenic properties. The simulation suggests that the B-cell epitope binds to B-cell receptors with remarkably high affinity, whereas the PrM–MAP vaccine induced a significant immune response in BALB/c mice. Finally, it was determined that this vaccine was completely safe and that there was no risk of tissue damage. In conclusion, the PrM–MAP design provides a promising alternative for preventing dengue fever, which could lead to better global health.

## 1. Introduction

Dengue is one of the most significant mosquito-borne viral diseases worldwide, with an annual count of 390 million, of which~96 million manifest clinically [1]. Dengue remains a major challenge because it has been categorized as one of the “neglected tropical diseases” by the World Health Organization (WHO), highlighting the discrepancy between its substantial burden and scant consideration in global health programmes [2]. Dengue is estimated to be responsible for 20,000 to 25,000 fatalities annually, predominantly among children, and remains endemic and widespread in over 100 countries [3]. Dengue is caused by four distinct yet closely related viruses (DENV-1 to DENV-4) belonging to the Flavivirus genus of the *Flaviviridae* family. The dengue virus is transmitted by mosquitoes *Aedes aegypti* and *A. albopictus*, and possesses a single-stranded, positive-sense RNA genome that encodes three structural (capsid, premembrane, and envelope) and seven non-structural (NS1, NS2a, NS2b, NS3, NS4a, NS4b, and NS5) proteins that are essential for viral replication and assembly [4]. Traditionally, the disease has been categorized into two primary clinical forms: dengue fever and the more severe dengue haemorrhagic fever/dengue shock syndrome (DHF/DSS). The World Health Organization (WHO) has revised the dengue classification system to improve clinical diagnosis and management. The traditional classification of dengue fever (DF), dengue haemorrhagic fever (DHF), and dengue shock syndrome (DSS) has been replaced by a simplified framework of the WHO that classifies dengue either with or without signs and severe dengue [5].

Despite years of research, a viable antiviral treatment for dengue is currently unavailable, highlighting the need for effective preventive measures. Moreover, the development of antiviral agents is a tedious process, and viral evolution may lead to drug-resistant strains [6]. While there are no effective antiviral drugs against dengue, vaccine development is the most effective approach to overcome the virus and to provide long-term immunity. Dengvaxia, a live attenuated, tetravalent, chimeric vaccine, has a yellow fever 17D virus backbone, genetically engineered to express the premembrane and envelope proteins of the four DENV serotypes, administered in three doses (0, 6, and 12 months) in individuals aged 9–16 years. A critical limitation is its recommendation exclusively for those with prior dengue exposure, as administration to seronegative individuals may enhance the risk of severe dengue upon subsequent infection [7,8]. TAK-003, another live attenuated tetravalent vaccine, shows strong protective efficacy against DENV-2, including in seronegative individuals, though its efficacy is notably lower against DENV-3 and DENV-4 in some populations [9]. The vaccine is based on the DENV-2 backbone, containing a native DENV-2 component, and expresses the PrM and envelope proteins of DENV-2, -3, and -4 to generate a broad immune response. The vaccine is administered in two doses for individuals in the age group of 4–16 years in high-transmission areas [10].

A crucial step in the understanding of dengue viral immunology is to identify and characterize B-cell epitopes that play a vital role in infection dynamics, disease pathogenesis, and the design of effective vaccines, as well as tools for diagnosis [11]. Epitope mapping of B-cell epitopes from structural and non-structural dengue viral proteins has also confirmed their diverse potential and immunological significance [12,13,14]. However, fewer have been investigated in the structurally conserved capsid (C) and premembrane/membrane (PrM/M) proteins [15,16,17].

The PrM protein is important for viral maturation as it acts as a molecular chaperone, stabilizing the envelope protein and helping it fold correctly [18]. From an immunological perspective, the premembrane protein (PrM) contains B-cell epitopes that can produce neutralizing antibody responses.

Although PrM-specific antibodies generally exhibit limited neutralizing activity and have been associated with antibody-dependent enhancement (ADE), the protein remains immunologically relevant because it contributes to the overall antigenic determinants of DENV [19,20]. A careful evaluation of PrM-derived epitopes could help find areas that trigger immune responses and broaden the target range for dengue vaccines.

The multiple antigenic peptide (MAP) system was employed to enhance epitope presentation for increased display because of its ability to elicit specific immune responses independent of traditional carrier proteins [21,22,23]. Although MAPs are mainly processed through the MHC class II pathway, research has demonstrated their ability to be taken up and transported through the MHC class I processing pathway as well, expanding their capabilities to induce both branches of the adaptive immune response [24].

The PrM protein sequences of 20 Pakistani dengue virus isolates, obtained from our previous study [16], were computationally analyzed to determine the most immunogenic B-cell epitope. Tetrameric PrM–MAP, carrying four copies of the identified B-cell epitope, was then evaluated in this study for its ability to elicit a humoral immune response and further evaluated for its safety as a vaccine candidate in Balb/c mice. By using both in silico predictions and in vivo analysis, this study will provide information that may be useful toward future applications of PrM-derived MAP constructs in dengue vaccine development.

## 2. Materials and Methods

### 2.1. Dengue PrM Sequence and B-Cell Epitope Selection

The choice of B-cell epitope from the dengue PrM protein consensus sequence was based on methodology previously reported by the same group [16]. The study involved the polyprotein sequences of the dengue virus serotypes prevalent in Pakistan, which were retrieved from GenBank (https://www.ncbi.nlm.nih.gov, accessed on 13 June 2022) and analyzed for their physicochemical properties, antigenic capability, structural topology, and homology in hosts. Consensus sequences were generated in silico, followed by predicting linear B-cell epitopes. The epitopes with the greatest number of MHC-I and MHC-II epitopes were evaluated for their immunogenicity, antigenicity, strain conservation, lack of toxicity, and allergenicity. Subsequently, the most immunogenic and safe B-cell epitope was selected for inclusion in the vaccine design.

### 2.2. In Silico Cytokine-Induction Analysis for PrM Epitope

To evaluate the potential of the selected B-cell epitope to induce a cytokine response, it was analyzed using immunoinformatics tools. Online servers IFNepitope (https://webs.iiitd.edu.in/raghava/ifnepitope/, accessed on 5 June 2026), IL4Pred (https://webs.iiitd.edu.in/raghava/il4pred/, accessed on 5 June 2026), IL-6Pred (https://webs.iiitd.edu.in/raghava/il6pred/, accessed on 5 June 2026), IL-10Pred (http://crdd.osdd.net/raghava/il10pred/, accessed on 5 June 2026) and TNF-α (https://webs.iiitd.edu.in/raghava/tnfepitope/, accessed on 5 June 2026) were used to characterize the epitope as an inducer or non-inducer of interferon-gamma (IFN-γ), interleukins IL-4, IL-6, and IL-10, and tumour necrosis fector-alpha (TNF-α), respectively. The analysis was conducted using the default settings provided by the servers.

### 2.3. PrM Sequence and B-Cell Epitope Selection

The 3D structure of the selected B-cell epitope was predicted using the PEP-FOLD4 online server (https://bioserv.rpbs.univ-paris-diderot.fr/services/PEP-FOLD4/, accessed on 13 January 2026). PEP-FOLD4 uses a coarse-grained representation of peptides, provides five top-ranked tertiary structure models, and has demonstrated reliable performance for both structures and highly charged peptides [25]. Subsequently, the highest-ranked model was selected for further analyses.

### 2.4. Epitope–Receptor Docking Analysis

The selected B-cell epitope was evaluated for its interaction with murine and human BCRs (PDB ID: 8EMA and PDB ID: 5IFH). Their PDB structures were retrieved from the RCSB Protein Data Bank (https://www.rcsb.org/, accessed on 10 January 2026) and optimized for docking using Discovery Studio Visualizer version 2021 (Dassault Systèmes BIOVIA, San Diego, CA, USA). Docking of the epitope to each receptor was performed by the ClusPro 2.0 server (https://cluspro.bu.edu/, accessed on 13 January 2026) using a rigid body docking approach that clusters energetically favourable conformations to predict probable binding orientations [26]. Cluster size and minimum energy values were considered, and the top-ranked docked complexes were selected for interaction analysis.

### 2.5. Receptor–Epitope Interactions

The selected docked structures were subsequently analyzed using the PDBsum Generate server (http://www.ebi.ac.uk/thornton-srv/databases/pdbsum/Generate.htmL, accessed on 14 January 2026) to characterize receptor–epitope interactions, highlighting hydrogen, disulfide, and non-bonded contacts.

### 2.6. MAP Structure Design and Physicochemical Characterization

The structural model tetrameric PrM–MAP-4 molecule incorporating the PrM epitope (AYTIGTTYFQRVLIFI) was generated using ChemSketch Freeware (Advanced Chemistry Development, Inc. Labs, Toronto, ON, Canada). The software was also used to evaluate the physicochemical characteristics of the designed vaccine.

### 2.7. Synthesis of Multiple Antigenic Peptides

The custom peptide vaccine, engineered as a lysine-core MAP-4 system, carrying four copies of the B-cell epitope, was commercially synthesized by LifeTein^®^ (LifeTein LLC; Somerset, NJ, USA). The purity and identity of the synthesized construct were analyzed by the manufacturer using reverse-phase high-performance liquid chromatography (RP-HPLC) with a C18 column and confirmed by mass spectrometry (MS).

The lyophilized MAP construct was reconstituted in phosphate-buffered saline (PBS) to prepare a 1 mg/mL stock solution without the addition of any adjuvants or other additives.

### 2.8. Animal-Based Experimental Analysis

All immunization experiments were performed at the Laboratory Animal House at Atta ur Rahman School of Applied Biosciences (ASAB), National University of Sciences and Technology (NUST), Islamabad, Pakistan. Animal handling and procedures were performed following the guidelines of the Institute of Laboratory Animal Research, National Institute of Health (NIH), USA rulings (NIH, 2011). Institutional ethical clearance was obtained from the IRB (Internal Review Board) of ASAB, NUST, with approval No. IRB-09-2023-ASAB-01/01.

A total of twenty-five 4–6-week-old male BALB/c mice were acquired from the National Institute of Health, Islamabad, Pakistan, and randomly divided into five groups (*n* = 5 per group) as follows: PBS control (CG 1), adjuvant control (CG 2), and three experimental groups (EG 1–EG 3). The experimental animals were differentiated based on the number of booster doses administered. Mice in the experimental groups received 50 µg of PrM–MAP by intraperitoneal injection in a final volume of 200 µL. The prime vaccine dose was administered on day 0, emulsified in Complete Freund’s adjuvant (CFA), followed by booster doses emulsified in Incomplete Freund’s adjuvant (IFA) on days 14, 35, and 56. Control group 2 received 50 µL of the adjuvant alone on the same schedule, while control group 1 was administered with 200 µL of phosphate-buffer saline (PBS).

Serum and organ samples were collected from EG 1 on day 28, EG 2 on day 49, EG 3 on day 71, and both control groups on day 71. All serum samples were stored at –20 °C until further use.

### 2.9. Detection of Preliminary Humoral Response Through Serum Enzyme-Linked Immunosorbent Assay (ELISA)

The immune response against PrM–MAP was assessed through an indirect ELISA method adapted from previously validated MAP vaccine protocols designed for the dengue virus [27] and infectious bronchitis virus [28].

PrM–MAP was coated onto 96-well microplates (Citotest Scientific Co., Ltd., Nanjing, Jiangsu, China) at a concentration of 5 µg/mL in 50 mM carbonate coating buffer (pH 9.6) and incubated overnight at 4 °C. Plates were washed three times with PBS containing 0.05% Tween-20 (PBST) and blocked with PBST supplemented with 3% bovine serum albumin (BSA) for 1 h at 37 °C. Serum samples from each group were diluted 1:50 in PBST/BSA, and 100 µL was added to each well, followed by incubation at 37 °C for 2 h.

After washing, 100 µL of horseradish peroxidase (HRP)-conjugated anti-mouse IgG (Applied Biological Materials Inc., Richmond, BC, Canada, diluted 1:5000) was added to each well, and the plates were incubated for 1 h at 37 °C. Following another washing, TMB (Applied Biological Materials Inc., Richmond, BC, Canada) was added, and the plates were placed in the dark for 20 min. Later, the reaction was stopped by adding 0.1 M H_2_SO_4_ (100 µL per well), and the absorbance at 492 nm was evaluated using an ELISA reader (BIO-RAD Tech. Watford, Hertfordshire, UK). The serum samples were analyzed in duplicates, and the mean absorbance values were taken for downstream statistical analysis.

### 2.10. Optimization of the Antigen Coating Concentration

To determine the effect of the MAP coating concentration on ELISA performance, PrM–MAP was coated onto microtiter plates at concentrations of 2, 3, and 4 µg/mL in addition to the standard coating density of 5 µg/mL. This optimization approach was conducted to evaluate assay sensitivity and signal discrimination at different antigen coating concentrations instead of investigating biologically dose-dependent effects.

### 2.11. Cell Viability Assay

The cytotoxic effect of the PrM–MAP construct was assessed using the MTT [3-(4,5-dimethylthiazol-2-yl)-2,5-diphenyltetrazolium bromide] assay, a well-established approach for assessing cell viability based on metabolic activity. HEK293 (human embryonic kidney) cells were selected for this experiment because of their predictable proliferative behaviour and excellent transfection efficiency. In addition, this was a human cell line that has been well established to screen for initial non-specific cytotoxicity. The HEK293 cell line was seeded in replicates, 8 × 10^3^ cells/well for the 48 h assay, into 96-well plates (NEST Biotechnology Co., Ltd., Wuxi, Jiangsu, China) containing Dulbecco’s modified Eagle medium (DMEM) supplemented with 10% fetal bovine serum (FBS) and 1% Pen/Strep, followed by incubation at 37 °C in a humidified atmosphere containing 5% CO_2_ for 24 h to allow proper cell attachment before treatment. For the initial step in the treatment, the old medium was replaced with fresh culture medium supplemented with varying concentrations of PrM–MAP (5, 10, 25, 50, 75, 100, 125, and 200 μg/mL) to study its cytotoxic effect at different doses. After 48 h of incubation, 15 µL of MTT solution (5 mg/mL) was added to each well and left for a further 3 h to develop formazan crystals. The medium was replaced with 100 μL of DMSO, and the plate was placed at 37 °C for 30 min to solubilize formazan crystals. DMSO-treated wells were used as the cytotoxicity control. The following formula was used to determine cell viability:

$$\begin{array}{cc} Viability(\%)= & Optical\;density\;of\;treated\;cells\\ & /Optical\;density\;of\;untreated\;cells\times 100 \end{array}$$

### 2.12. Histopathological Investigations

The mice were euthanized on days 28, 49, and 71, and the liver, brain, spleen, and kidney were aseptically excised from the animals’ bodies and later fixed in 10% neutral buffered formalin prepared in distilled water. Tissues were later processed and embedded in paraffin, sectioned at 5 micrometre thicknesses, and stained with haematoxylin and eosin (H&E) for histopathological examination [29]. Tissue sections were evaluated through histopathological analysis using a light microscope, (OPTIKA B-150 biological microscope (OPTIKA S.r.l., Via Rigla 30, 24010 Ponteranica (Bergamo Province), Italy) and representative images were captured at both 4× and 10× magnification.

### 2.13. Statistical Evaluation

All statistical analyses were conducted using GraphPad Prism 8.0 (San Diego, CA, USA). Differences in the ELISA results among the study groups were evaluated using the non-parametric Kruskal–Wallis’s test. Cell viability assay data were analyzed using one-way analysis of variance (ANOVA). A *p*-value < 0.05 was considered statistically significant. Cut-off values for ELISA were calculated from the OD492 values of the negative control (CG1) for a 95% confidence interval using the following formula:

$$\mathrm{Cut-off}=X^{-}+SDf$$

where X^−^ and SD represent the mean absorbance of the negative controls and the corresponding standard deviation, respectively.

## 3. Results

### 3.1. Epitope Selection

The B-cell epitope, used as an antigenic peptide in MAP, was selected based on a previous study, which identified conserved and immunodominant regions within the PrM protein of the dengue virus isolates prevalent in Pakistan [16]. Among the shortlisted candidates, the PrM-derived “AYTIGTTYFQRVLIFI” showed the highest immunogenic score and an antigenic value above the threshold. The epitope demonstrated 100% conservancy among all dengue isolates of serotype 2, whereas 56% and 60% conservancy was observed in the DENV-4 and DENV-3 isolates, respectively.

### 3.2. Predicted Cytokine-Inducing Profile for PrM Epitope

The selected B-cell epitope was computationally evaluated for its capability to induce cytokines. The peptide was predicted to have the potential to induce IFN-γ, IL-4, IL-6, and IL-10 with prediction scores of 0.073, 0.27, 0.23, and 0.99, respectively. The epitope was found to be a non-inducer of TNF-α. Thus, the results suggested that the PrM epitope may possess the capacity to stimulate both immune-activating and immunoregulatory pathways, providing additional support for the observed humoral response in the subsequent in vivo studies (Table 1).

### 3.3. Structural Modelling of the Predicted B-Cell Epitope Structure

**Figure 1:**
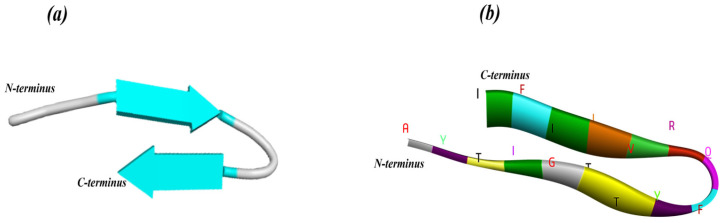
Predicted three-dimensional structure of the B-cell epitope from PrM dengue viral protein with PEP-FOLD4. (**a**) Secondary structure with β-strands (cyan arrows, directed from N to C terminus) and coils (grey). (**b**) Predicted 3D model showing amino acid residues alanine and glycine (grey), tyrosine (violet), threonine (yellow), arginine (red), isoleucine (green), glutamine (magenta), valine (light green), leucine (orange), and phenylalanine (cyan).

In silico structural modelling of the PrM epitope could serve as a reliable foundation for subsequent studies of molecular interactions. The tertiary structure of the PrM-B-cell epitope sequence (AYTIGTTYFQRVLIFI), predicted via PEP-FOLD4, revealed two beta sheets (cyan arrows) connected by an intervening coil region (grey), showing a defined spatial organization of the peptide (Figure 1a).

The ribbon representation also illustrated the overall topology of the epitope, revealing a loop-dominated structure with no prominent α helices (Figure 1b). The predicted model provided a structural framework for subsequent immunoinformatics and docking analysis.

### 3.4. Epitope–Receptor Docking and Interaction Study

A molecular docking analysis was conducted to assess the interactions of the selected B-cell epitope (purple) with murine BCR (PDB ID: 8EMA) and the human BCR Fab region (PDB ID: 5IFH) to obtain a structural assessment of its potential recognition by BCRs.

#### 3.4.1. Structural Characterization of PrM-Epitope-Human BCR Fab Region (PDB ID: 5IFH) Complex

**Figure 2:**
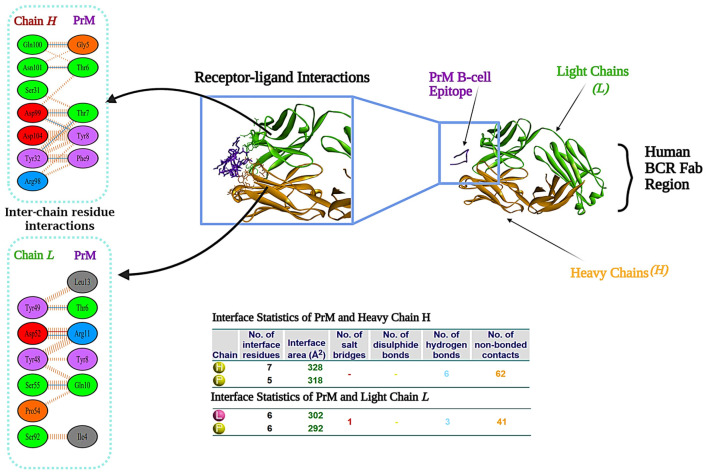
Predicted inter-chain residue interaction of PrM-B-cell epitope (in purple) with the human BCR Fab region (PDB ID: 5IFH), showing the binding interface, interacting residues, and interface statistics. The light chain of the antibody is represented in green, and the heavy chain is in orange, constituting the Fab region of the antibody. The colours used to denote the type of inter-chain inter-actions are identical in the figure and interface statistics table.

Molecular docking analysis revealed favourable interactions between the PrM-B-cell epitope and the human BCR Fab region (PDB ID: 5IFH). The residues from both light and heavy chains contributed to epitope recognition through six hydrogen bonds and 62 non-bonded contacts with the amino acid residues Glycine (Gly) 5, Threonine (Thr) 7, Thr 8, and Phenylalanine (Phe) 9 of the epitope (Figure 2). In conclusion, the participation of both receptor chains’ structural complementarity underscored the potential recognition of the PrM epitope by the human BCR.

#### 3.4.2. Structural Characterization of the PrM-Epitope-Mouse BCR (PDB ID: 8EMA) Complex

**Figure 3:**
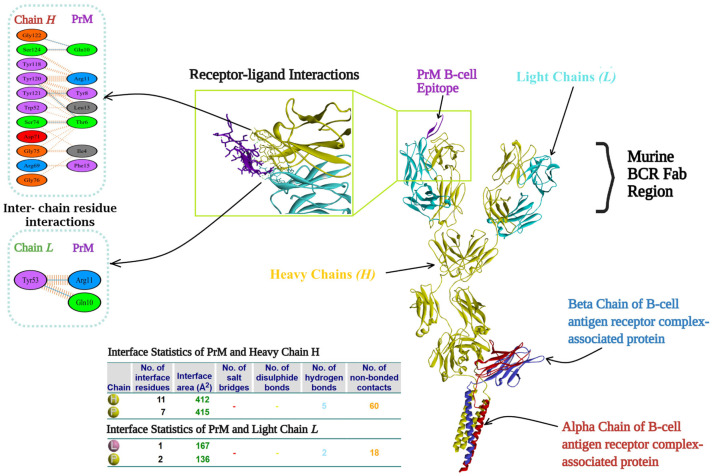
*Inter-chain* interaction pattern between the PrM B-cell epitope (purple) and Mouse BCR (PDB ID: 8EMA), highlighting the binding pocket, interface statistics, and interacting amino acids of the PrM epitope and the light (cyan) and heavy (yellow) chains. The colours used to denote the type of inter-chain inter-actions are identical in the figure and interface statistics table.

Structural characterization of the docked BCR-epitope complex revealed substantial contacts between the PrM epitope and the antigen-binding cleft. The majority of these interactions involved residues from the heavy chain; however, only one residue (Tyr 53) from the light chain contacted two residues (Arg 11 and Gln 10) of the epitope (Figure 3).

Quantitative analysis revealed significant binding structures, with the heavy chain forming five hydrogen bonds and many non-bonded interactions. The light chain also contributed to the stability by forming two hydrogen bonds and 18 non-bonded interactions. Collectively, these multiple stabilizing contacts and good interface geometry indicated a notable binding of the PrM epitope to the BCR without disturbing the transmembrane alpha and beta chains of the receptor.

### 3.5. Design of MAP Architecture and Physicochemical Characteristics Evaluation

**Figure 4:**
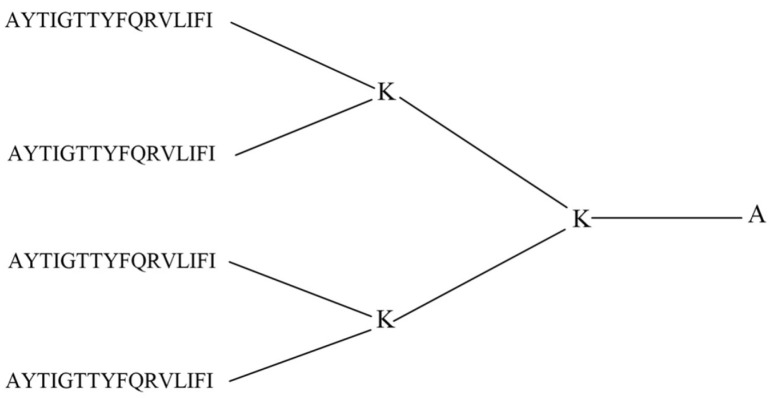
Structural design of PrM–MAP-4 construct showing four copies of PrM epitope arranged on a branched lysine core for antigen presentation.

Using ACD/ChemSketch freeware, the PrM–MAP structure was designed in MOL format and later subjected to physicochemical analysis. The construct had a core of three lysine (K) residues, initiating from an alanine (A) residue, with four identical sequences (AYTIGTTYFQRVLIFI), conjugated to ε-amino groups of terminal lysine residues (Figure 4). The dendritic structure enables the multivalent display of epitopes that might mimic the virus particle and is related to improved B-cell recognition and enhanced immunogenicity in the MAP-based vaccine approach.

**Figure 5:**
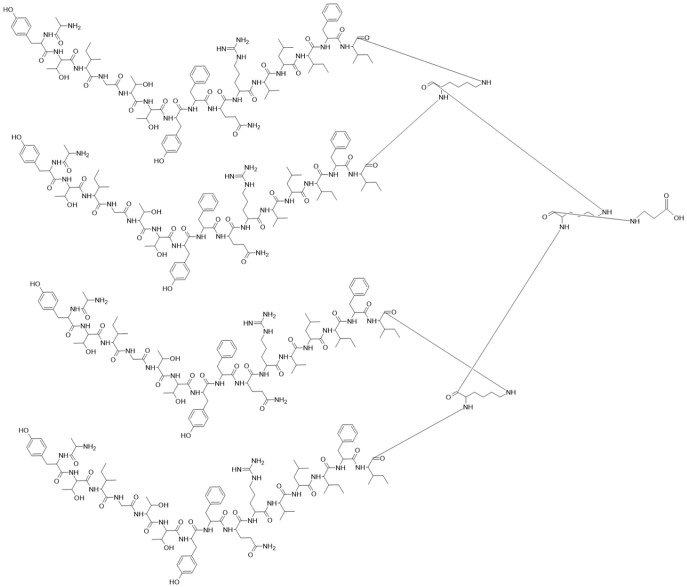
Two-dimensional molecular structure of the synthesized PrM–MAP molecule generated via ChemSketch Freeware, highlighting the lysine-core branching approach used to display four copies of the PrM epitope.

The lysine-based dendrimer enabled multimeric antigens while minimizing the need for carrier proteins. The chemical structure of the tetrameric MAP constructs displayed the spatial arrangement of four copies of the dengue virus PrM-derived peptide covalently linked to a lysine-based branched core (Figure 5).

The physicochemical characteristics of PrM–MAP were assessed using ChemSketch. The construct had a molecular formula of C_393_H_595_N_87_O_93_ and a molecular weight of 8026.5990 Da, reflecting the large size and chemical complexity of the tetrameric peptide assembly.

The elemental composition of the molecule showed a ratio of carbon, nitrogen, and oxygen content of 58.81%, 15.18%, and 18.53%, respectively. Moreover, a molar refractivity of 2089.49 ± 0.5 cm^3^ and a molar volume of 5800.5 ± 7.0 cm^3^, which are consistent with the extensive three-dimensional conformation, were also demonstrated. The high Parachor value of 15,975.8 ± 8.0 cm^3^ and polarizability of 828.34 ± 0.5 10^−24^ cm^3^ are further consistent with a bulky, highly interactive molecular surface, which is pertinent for epitope recognition and antigen presentation (Table 2).

The optical properties, along with an index of refraction of 1.639 ± 0.05, showed that the dense structural framework was reinforced. Furthermore, the construct also exhibited a surface tension of 57.5 ± 7.0 dyne/cm, and with a density of 1.38 ± 0.1 g/cm^3^, there was an indication of moderate solubility and compactness. In addition, a ring double-bond equivalent (RDBE) of 140 proves unsaturation and complexity (Table 2). The overall physicochemical characteristics confirmed that PrM–MAP exhibits a compact and interactive molecular structure, reflecting favourable molecular properties and structural stability.

The HPLC results with a C18 column showed a significant peak at a retention time of 12.84 min, representing a purity level of 95.43%, confirming a successful synthesis process with little contamination (Supplementary File S1). The identity of the construct was further confirmed by MS analysis, yielding a molecular mass that was consistent with the expected PrM–MAP (Supplementary File S2).

### 3.6. Serum Enzyme-Linked Immunosorbent Assay (ELISA) for the Detection of Preliminary Humoral Response in Balb/C Mice

To assess the immune response induced by the PrM–MAP vaccine, 25 mice were randomly distributed into five groups (*n* = 5 per group). Control group 1 received PBS, whereas control group 2 received the adjuvant alone. The three experimental groups received an identical PrM–MAP immunization protocol consisting of a prime dose with CFA on day 0 and booster doses with IFA on days 14, 35, and 56. The experimental groups were taken as “endpoint cohorts” to collect the samples at a predetermined time, but they did not receive any different treatment. Experimental group 1 was euthanized and sampled on day 28, experimental group 2 on day 49, and experimental group 3 on day 71. Both control groups were sampled on day 71 (Table 3). Serum samples were collected at designated intervals, and mice were euthanized two weeks following the final dose to assess peak antibody production, aligning with vaccine response timelines and B-cell maturation [30].

**Figure 6:**
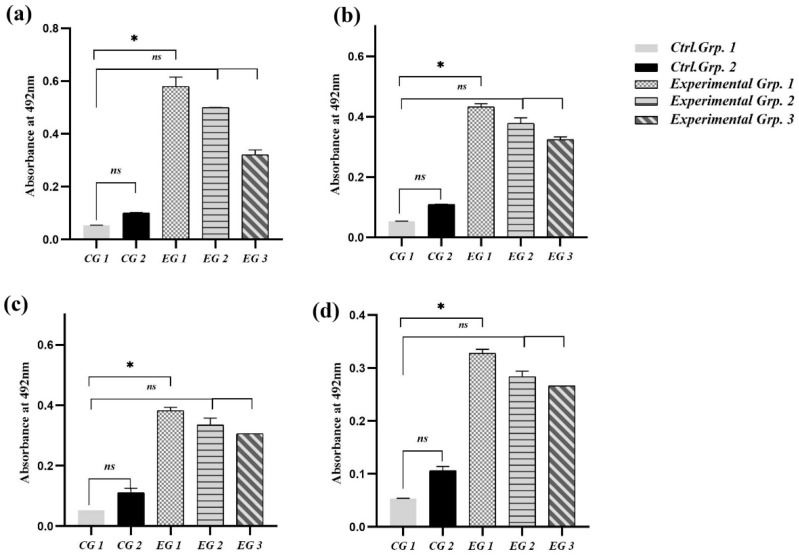
Humoral immune response induced by PrM–MAP vaccine in Balb/c mice evaluated by indirect ELISA. Serum immunoglobulin reactivity (A492) measured at varying antigen coating concentrations: (**a**) 2 µg/mL; (**b**) 3 µg/mL; (**c**) 4 µg/mL; (**d**) 5 µg/mL. Control groups received PBS (CG1) or adjuvant alone (CG2), while experimental groups (EG1–EG3) were administered one, two, or three booster doses. Statistical analysis was performed via the Kruskal–Wallis test; *p* < 0.05 was taken as significant. *p* > 0.05 (ns/non-significant), *p* < 0.02 (*).

Indirect ELISA revealed a detectable antigen-specific response in all PrM–MAP-immunized groups (EG1, EG2, and EG3) compared with the control groups (CG1 = PBS and CG2 = adjuvant only) across all antigen coating concentrations. The highest mean absorbance values were observed for EG1, which exhibited a statistically significant antibody response compared with the PBS control (*p* < 0.05). Although the absorbance values for CG1 and CG2 were higher than those of the control groups, the responses were not statistically significant (Figure 6a–d). Antibody reactivity remained measurable throughout the study, but a gradual decrease at later time points was observed, indicating a persistent immune induction after PrM–MAP vaccination.

### 3.7. Effect of PrM–MAP Antigen Coating Concentration

**Figure 7:**
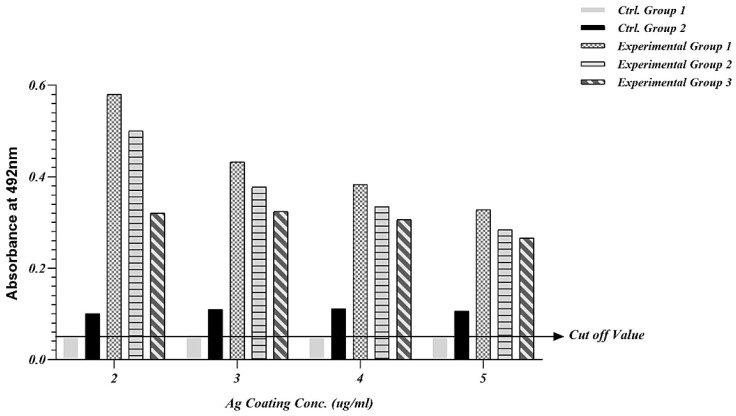
Cut-off-based assessment of ELISA performance at different PrM–MAP coating concentrations. Absorbance values from the same ELISA dataset shown in Figure 6 are replotted to evaluate the effect of the antigen coating concentration (2–5 µg/mL) on assay discrimination relative to the predefined cut-off value (0.05). Control groups received PBS (CG1) or adjuvant alone (CG2), while experimental groups (EG1–EG3) were administered one, two, or three booster doses. The cut-off value was evaluated as 0.05 ± 0.001, as the control values were almost constant.

The antigen coating concentration affected assay sensitivity and signal discrimination. At lower coating concentrations (2 and 3 µg/mL), positive samples were clearly separated from the cut-off value (0.05), highlighting enhanced differentiation between responsive and non-responsive sera. The higher antigen coating appeared to reduce the signal resolution. The adjuvant-only group showed absorbance values close to the cut-off value, indicating non-specific reactivity rather than an actual antigen-specific immune response (Figure 7). Collectively, these findings, consistent with the previous studies [28], indicate that optimizing antigen coating densities can improve ELISA sensitivity and help minimize non-specific background signals.

### 3.8. Evaluation of Cell Viability

**Figure 8:**
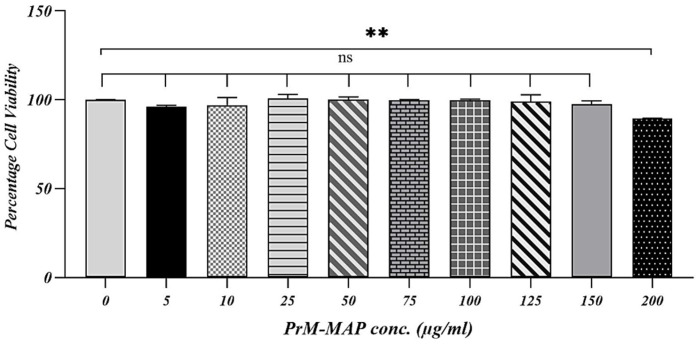
Cell viability analysis of PrM–MAP via MTT assay after 48 h of incubation at increasing antigen concentrations. The construct showed minimal cytotoxicity across all concentrations, with a significant decrease at 200 µg/mL. One-way ANOVA was employed, and *p* < 0.05 was taken as significant. *p* = 0.1000 (ns/non-significant), *p* = 0.002 (**).

The cytotoxicity effect of PrM–MAP was assessed in HEK293 cells using the MTT assay. Cell viability remained comparable to the control (untreated) across the tested concentrations (5–150 µg/mL). The viability values persisted close to 100% with a statistically non-significant difference (Figure 8). A slight but statistically significant reduction in cell viability was observed only at the highest concentration (200 µg/mL). In summary, the PrM–MAP construct exhibited minimal cytotoxicity over the tested concentration range, suggesting acceptable tolerability under the experimental conditions, thus supporting its future in vivo testing and safe vaccine development.

### 3.9. Histopathological Assessments

Histological analysis of the brain tissue did not show any treatment-related damage in either the experimental or control groups.

**Figure 9:**
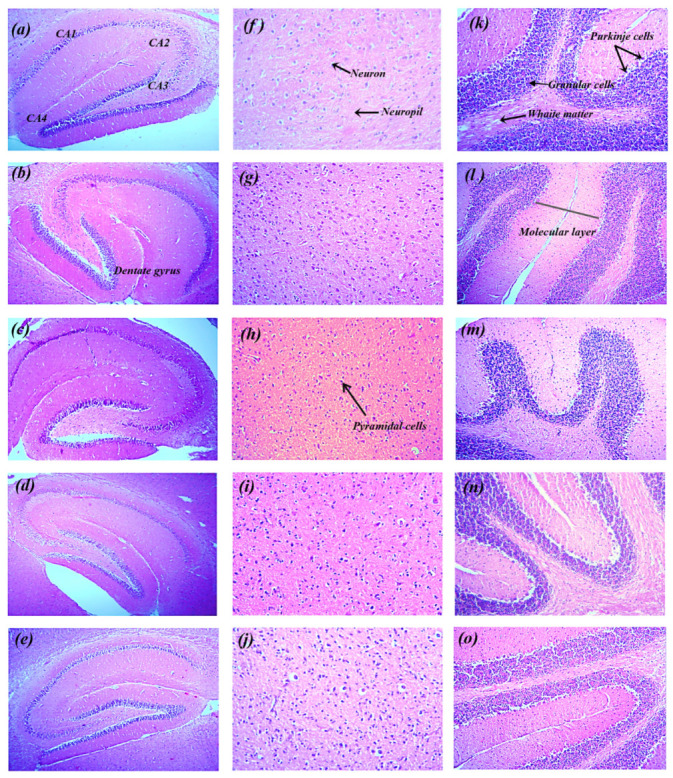
Representative photomicrographs of haematoxylin and eosin (H&E)-stained sections of mouse brain regions, including the hippocampus (4×), cerebral cortex (10×), and cerebellum (10×), collected at different time points after immunization with PrM–MAP. Panels (**a**,**f**,**k**) represent the PBS control group and panels (**b**,**g**,**l**) the adjuvant control group (day 71), panels (**c**,**h**,**m**) Experimental Group 1 (day 28), panels (**d**,**i**,**n**) Experimental Group 2 (day 49), and panels (**e**,**j**,**o**) Experimental Group 3 (day 71). Histopathological evaluation revealed normal brain tissue structure with no signs of cellular or organ architecture in the control and experimental groups.

The hippocampus at low magnification (4×) revealed an intact architecture, with well-preserved subregions CA1-CA3 and dentate gyrus, and no signs of lesions, necrosis, hemorrhage, or pathological changes were observed (Figure 9a–e).

Similarly, the cerebral cortex retained its normal laminar arrangement with no signs of neuronal degeneration, spongiosis, or any form of inflammatory cell infiltration (Figure 9f–j). Moreover, the cerebellum and basal ganglia showed no histological disruptions or damage (Figure 9k–o).

**Figure 10:**
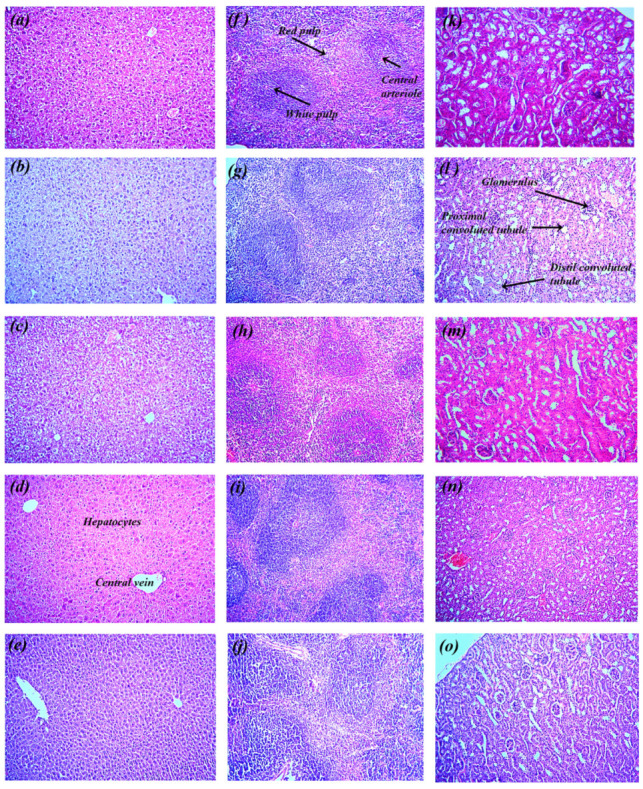
Representative photomicrographs at 10× of haematoxylin and eosin (H&E)-stained sections of mouse liver tissue (left column), spleen (middle column), and kidney (right column) collected at different post-immunization days. Panels (**a**,**f**,**k**) represent the PBS control group, panels (**b**,**g**,**l**) the adjuvant control group (day 71), panels (**c**,**h**,**m**) Experimental Group 1 (day 28), panels (**d**,**i**,**n**) Experimental Group 2 (day 49), and panels (**e**,**j**,**o**) Experimental Group 3 (day 71). Microscopic study of the organ tissues showed no signs of damage or necrosis in the control and experimental groups.

Upon histopathological evaluation, the liver tissues showed no signs of inflammation, necrosis, hepatocellular degeneration, or disruption of tissue architecture for any of the study groups. The preserved histological features of the hepatic lobules and central veins appeared unaffected, and no signs of liver injury were observed (Figure 10a–e).

Along the same lines, the splenic tissue showed a normal histological structure with well-preserved red and white pulp, with adequate vascular perfusion, and the surrounding lymphoid tissue. The tissue also had an adequate connective tissue trabecula with no inflammation or tissue damage (Figure 10f–j).

In addition, the renal tissue displayed normal morphology, intact glomeruli and convoluted tubules, and an overall healthy cellular organization. No evidence of inflammation, structural abnormality, or degenerative lesions was found (Figure 10k–o).

In conclusion, the preservation of normal histoarchitecture in the brain, liver, spleen, and kidney implied that PrM–MAP was well tolerated under the tested doses. Thus, it provides initial safety evidence to justify its evaluation for further preclinical development.

## 4. Discussion

Multiple antigenic peptide (MAP) systems allow multimeric antigens without conventional carrier proteins and have therefore been widely studied for their potential applications in the design of peptide vaccines [15,31]. Also, there is no risk of reverting the pathogen to the virulent form. In addition to enhancing immunological recognition, the lysine core of the MAPs suppresses the unwanted immune responses that are typically caused by carrier proteins [32,33,34].

In the current study, a B-cell epitope of the dengue virus PrM protein was incorporated into a MAP construct, and it was tested through complementary in silico and in vivo methods. Although the B-cell epitope composition of multiple dengue virus proteins has been explored [35], the PrM region is relatively under-investigated. These findings favour the potential of the PrM region as a candidate target for peptide-based dengue vaccine development. The immunogenicity of the epitope derived from PrM observed here may be due to its surface accessibility during maturation of the virus and the ability to present antigenic determinants that are recognized by B-cell receptors. While the envelope (E) protein has been the primary target for dengue vaccine development, increasing evidence indicates that accessory structural proteins such as PrM may contribute to dengue-specific humoral responses and expand the antigenic repertoire for vaccine design [36,37].

Cytokine prediction analysis provided additional computational support for the relevance of the selected PrM-derived epitope from an immunological perspective. The capacity to induce IFN-γ may possibly contribute to the activation of the cellular immune system, while the ability to induce IL-4 can support B-cell differentiation and generation of antibodies, correlating with the humoral immune response observed in the following PrM–MAP immunization experiment. The propensity of the epitope to induce IL-6 may be associated with immune cell activation and maturation in the initial stages of the immune reaction. In addition, the most prominent prediction relates to IL-10 induction, a cytokine involved in the regulation of inflammation during antigen presentation. However, the epitope was predicted to be non-inducer of TNF-α, indicating a lower potential to induce a pro-inflammatory response. Similar cytokine-oriented bioinformatics approaches have been applied in the development and evaluation of vaccines against flaviviruses, including dengue and Zika viruses, where the possibility to induce IL-4, IL-10, and IFN-γ was used to support epitope selection [38,39]. Nevertheless, the results should be interpreted cautiously, as the predicted cytokine functionality provided supplementary computational evidence and not a confirmation of biological activity.

The observed contacts between the epitope and both mouse and human BCRs offer a plausible structural reason for the detected humoral responses. These in silico studies were meant to complement epitope accessibility and not predict functional outcomes. Positive interactions observed with both murine and human BCR models support the structural accessibility of the epitope and are consistent with the antigen-specific humoral responses observed in vivo [40].

The observed humoral response showed that the MAP approach effectively presented the PrM-derived B-cell epitope to the adaptive immune system. The antibody kinetics pattern in this experiment, which started high and then slightly decreased, was comparable to the physiological immune contraction following successful priming. Repeated antigen exposure may trigger feedback regulatory mechanisms that stabilize humoral responses rather than continuously increase antibody production [41]. This has been demonstrated in the case of peptide-based vaccination, where repeated exposure typically resulted in a response following the initial expansion phase rather than an increase in humoral responses [32,42].

The enhanced discrimination of antigen-specific signals at lower concentrations of MAP coating also coincides with the principles of ELISA optimization, where a high antigen density may lead to increased non-specific binding and steric hindrance, enhancing the background and reducing sensitivity [28]. In this connection, the higher signal discrimination at lower coating concentrations may indicate better performance of the assay signal’s clarity, but not immunogenicity differences. These findings highlight the importance of assay optimization for the assessment of vaccine-induced immune response, as an inappropriate antigen coating concentration may underestimate immunoglobulin binding capacity and possibly hinder biologically important differences between the study groups [43].

The safety tests, including histopathological studies and MTT-based cytotoxicity studies, revealed that the PrM–MAP construct could be tolerated at the concentration relevant to immunological testing [44,45]. The plausible reason for the favourable safety profile of MAP-based vaccines is their chemically defined nature, which is devoid of infectious components and does not require the use of live or attenuated pathogens. Moreover, peptide-based formulations typically show lower risks of systemic toxicity and off-target effects because of their high specificity and predictable breakdown in the body [46]. While not a synthetic polymeric dendrimer structure for drug delivery, the safety profile of MAP constructs should also be an important consideration for clinical translation. Recent advances in dendrimer engineering demonstrate that their biosafety is quite achievable by rational molecular design, such as size, surface properties, biodegradability and biocompatibility [47]. These principles can also help guide future optimization and clinical development of MAP based vaccine platforms, alongside the in vivo and in vitro results of the present study.

Although the PrM–MAP vaccine induced a measurable antigen-specific humoral response in vivo and maintained a suitable safety profile, certain limitations are important to consider. In addition to the small sample size, antibody titres were measured at a single serum dilution, and the functional properties of the generated antibodies, like neutralizing capacity and protective relevance, were not assessed. In addition, cellular immune responses and cytokine profiles were not investigated. Future studies should include an assessment of the antibody titres, virus-neutralization assays, cytokine profiling, and flow cytometric analyses, as well as a larger sample size, to provide a more comprehensive characterization of the immune responses elicited by the PrM–MAP construct and to support its further preclinical development.

## 5. Conclusions

This study demonstrated that a dengue virus PrM-derived B-cell epitope, screened through immunoinformatic analysis, can be effectively incorporated into a MAP construct. The resulting PrM–MAP vaccine induced a detectable antigen-specific humoral response in Balb/c mice while exhibiting a favourable safety profile in both cytotoxicity and histopathological evaluation. The alignment between computational prediction and experimental outcomes supported the utility of an immunoinformatic-guided MAP approach for peptide vaccine design. Collectively, these results highlight the ability of MAP-based epitope vaccines as a promising strategy for dengue vaccine research and provide a base for future studies to assess the protective efficiency and functionality of the antibodies and translational applicability.

## Figures and Tables

**Table 1:** In silico prediction of cytokine-inducing potential of the selected dengue virus PrM-derived B-cell epitope.

Cytokine	Predictive Score	Predicted Activity
IFN-γ	0.073	Positive inducer
IL-4	0.27	IL-4 inducer
IL-6	0.23	IL-6 inducer
IL-10	0.99	IL-10 inducer
TNF-α	-	Non-inducer

**Table 2:** Physicochemical characteristics of the PrM–MAP molecule evaluated via ChemSketch Freeware.

Physicochemical Properties	Values
Molecular Formula	C_393_H_595_N_87_O_93_
Formula Weight	8026.5990
Composition	C (58.81%) H (7.47%) N (15.18%) O (18.54%)
Molar Refractivity	2089.49 ± 0.5 cm^3^
Molar Volume	5800.5 ± 7.0 cm^3^
Parachor	15,975.8 ± 8.0 cm^3^
Index of Refraction	1.639 ± 0.05
Surface Tension	57.5 ± 7.0 dyne/cm
Density	1.38 ± 0.1 g/cm^3^
Polarizability	828.34 ± 0.5 10^−24^ cm^3^
RDBE	140
Monoisotopic Mass	8021.450392 Da
Nominal Mass	8017 Da
Average Mass	8026.599 Da

**Table 3:** Experimental design for mice immunization. (PrM–MAP concentration = 50 µg/200 µL of injection).

Groups	No. of Mice	Time of Immunizations				Time of Serum Collection
		Day 0	Day 14	Day 35	Day 56	
**EG 1**	5	MAP + CFA (200 µL)	MAP + IFA (200 µL)			Day 28
**EG 2**	5	MAP + CFA (200 µL)	MAP + IFA (200 µL)	MAP + IFA (200 µL)		Day 49
**EG 3**	5	MAP + CFA (200 µL)	MAP + IFA (200 µL)	MAP + IFA (200 µL)	MAP + IFA (200 µL)	Day 71
**CG 1**	5	PBS (200 µL)	PBS (200 µL)	PBS (200 µL)	PBS (200 µL)	
**CG 2**	5	Adjuvant CFA (50 µL)	Adjuvant IFA (50 µL)	Adjuvant IFA (50 µL)	Adjuvant IFA (50 µL)	

## Data Availability

The original contributions presented in this study are included in the article. Further inquiries can be directed to the corresponding author.

## Supplementary Materials

The following supporting information can be downloaded at: https://www.mdpi.com/article/10.3390/biology15141201/s1, File S1: HPLC report (LifeTein LLC; Somerset, NJ, USA); File S2: Mass Spectrometry Report (LifeTein LLC; Somerset, NJ, USA).

## Abbreviations

The following abbreviations are used in this manuscript:

MAP	Multiple antigenic peptide
PDB	Protein Data Bank
WHO	World Health Organization
DENV	Dengue virus
DF	Dengue fever
DHF	Dengue hemorrhagic fever
PrM	Premembrane
BCR	B-cell receptor
ADE	Antibody-dependent enhancement
MHC	Major histocompatibility complex
CFA	Complete Freund’s adjuvant
IFA	Incomplete Freund’s adjuvant
PBST	Phosphate-buffer saline Tween
BSA	Bovine serum albumin
TMB	3,3′,5,5′-tetramethylbenzidine
MTT	[3-(4,5-dimethylthiazol-2-yl)-2,5-diphenyltetrazolium bromide]
HEK	Human embryonic kidney cells
H&E	Haematoxylin and eosin
