# Supplementary material for: A Dendrimer-Based Multiple Antigenic Peptide (MAP) Approach for Dengue Vaccine Development: In Silico and In Vivo Insights on Safety and Effectiveness
Source: Biology (Basel). 2026 Jul 20;15(14):1201. doi: 10.3390/biology15141201 (PMC13405821; doi:10.3390/biology15141201)
Supplement: Supplementary file 1 [file biology-15-01201-s001.zip › biology-4347194-supplementary-proof/File S1-HPLC report (LifeTein LLC; new Jersey, USA).pdf]

Understanding Life
One Protein at a Time ...

HPLC REPORT

Sample Description:

Structure : #1 Peptide 1 AI-16

Number : 0200046

Lot # : LT230206-LT1050604

Column : 4.6mm*250mm, C18

Mobile Phase : A=0.1% TFA/Acetonitrile,

: B=0.1%TFA/water,

Gradient :	A	B
0.01min	20%	80%
25min	65%	35%
25.1min	100%	0%
30.0min	STOP	

Flow rate : 1.0ml/min

Wavelength : 220nm

Volume : 5ul

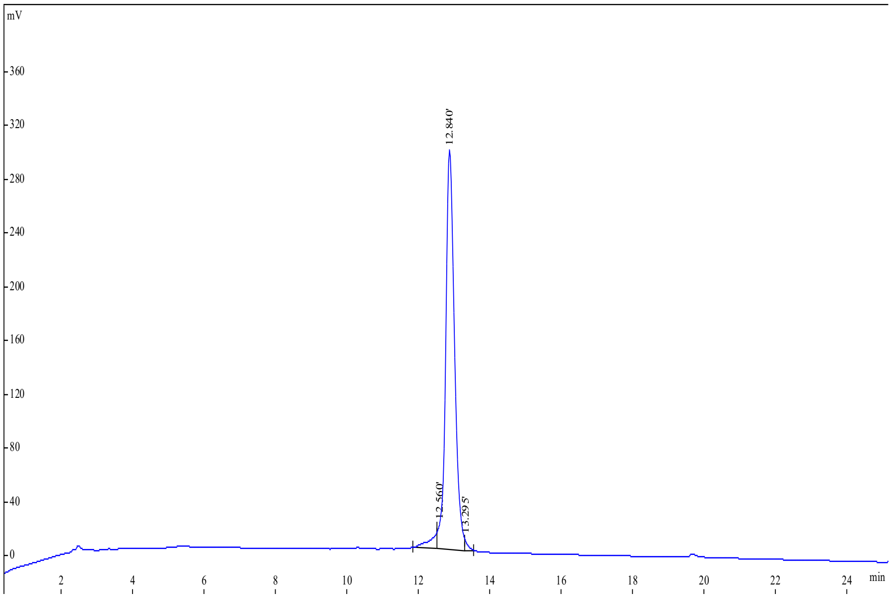

Rank	Time	Conc.	Area	Height
1	12.560	3.981	212859	19499
2	12.840	95.43	5101748	298633
3	13.295	0.5896	31522	6347
Total		100	5346129	324479

Tel: 1-732-444-3282 • Fax: 1-888-791-1618 • Email: info@lifetein.com
