# Supplementary material for: A Dendrimer-Based Multiple Antigenic Peptide (MAP) Approach for Dengue Vaccine Development: In Silico and In Vivo Insights on Safety and Effectiveness
Source: Biology (Basel). 2026 Jul 20;15(14):1201. doi: 10.3390/biology15141201 (PMC13405821; doi:10.3390/biology15141201)
Supplement: Supplementary file 1 [file biology-15-01201-s001.zip › biology-4347194-supplementary-proof/File S2-Mass Spectrometry Report (LifeTein LLC; new Jersey, USA).pdf]

# Mass Spectrometry Report

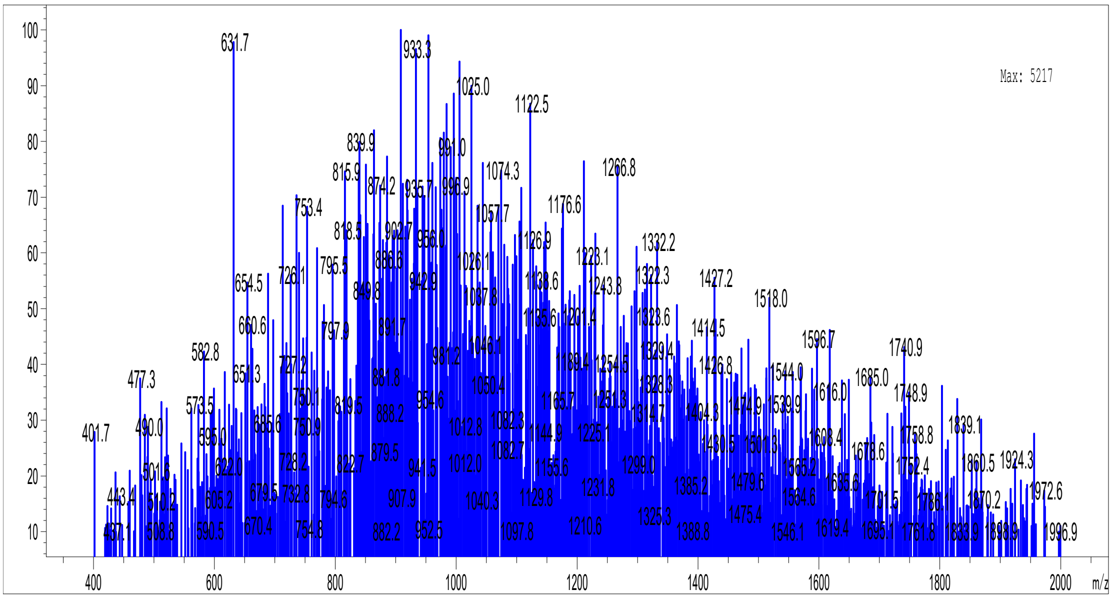


**Sample: 1050604** **MW: 8026.46**

| Item | Parameter | Item | Parameter |
|---|---|---|---|
| Probe: | ESI | Probe bias: | +4.5kv |
| Nebulizer Gas Flow: | 1.5L/min | Detector: | 1.5kv |
| CDL: | -20.0v | T.Flow: | 0.2ml/min |
| CDL Temp: | 250°C | B.conc: | 50%H2O50%ACN |
| Block Temp: | 200°C | | |
